# Imaging of human differentiated 3D neural aggregates using light sheet fluorescence microscopy

**DOI:** 10.3389/fncel.2014.00221

**Published:** 2014-08-06

**Authors:** Emilio J. Gualda, Daniel Simão, Catarina Pinto, Paula M. Alves, Catarina Brito

**Affiliations:** ^1^Cell Imaging Unit, Instituto Gulbenkian de CiênciaOeiras, Portugal; ^2^iBET - Instituto de Biologia Experimental e TecnológicaOeiras, Portugal; ^3^Instituto de Tecnologia Química e Biológica, Universidade Nova de LisboaOeiras, Portugal

**Keywords:** fluorescence microscopy, light-sheet fluorescence microscopy, 3D neural aggregates, calcium imaging, live/dead assays

## Abstract

The development of three dimensional (3D) cell cultures represents a big step for the better understanding of cell behavior and disease in a more natural like environment, providing not only single but multiple cell type interactions in a complex 3D matrix, highly resembling physiological conditions. Light sheet fluorescence microscopy (LSFM) is becoming an excellent tool for fast imaging of such 3D biological structures. We demonstrate the potential of this technique for the imaging of human differentiated 3D neural aggregates in fixed and live samples, namely calcium imaging and cell death processes, showing the power of imaging modality compared with traditional microscopy. The combination of light sheet microscopy and 3D neural cultures will open the door to more challenging experiments involving drug testing at large scale as well as a better understanding of relevant biological processes in a more realistic environment.

## Introduction

Human cellular models capable of mimicking the characteristics of living tissues are essential in both basic research and drug discovery. Cells within a tissue constantly integrate external cues that influence important cellular functions such as proliferation and differentiation, to which the interaction with neighboring cells and extracellular matrix is crucial. Thus, three-dimensional (3D) models present a more physiologically relevant approach that can increase the reliability and predictability of pre-clinical assays (Pampaloni et al., 2007; Breslin and O'Driscoll, 2013), while decreasing the dependence on animal testing in pharmaceutical industry. Additionally, 3D cellular aggregates represent a simple and straightforward strategy even for fundamental studies on pathological pathways in human disorders. Such aggregates have been used for a broad spectrum of studies in cancer biology to study proliferation, cell death, differentiation, and metabolism of cells in tumors and the response of tumors to radiotherapy and chemotherapy (Hirschhaeuser et al., 2010; Vinci et al., 2012). Also, *hepatocyte spheroids* have been proposed as a *cell* model for a variety of diagnostic, discovery, and therapeutic applications, such as a bio-artificial liver (No et al., 2012; Yu et al., 2012). As for central nervous system (CNS) modeling, 3D neural aggregates have been reported to efficiently mimic basic processes of brain development (Moors et al., 2009). Recently, human induced pluripotent stem cell-derived (iPSC) neurons were reported to self-organize in 3D cortical structures, recapitulating the early dorsal telencephalic developmental program (Mariani et al., 2012).

**Figure 1:**
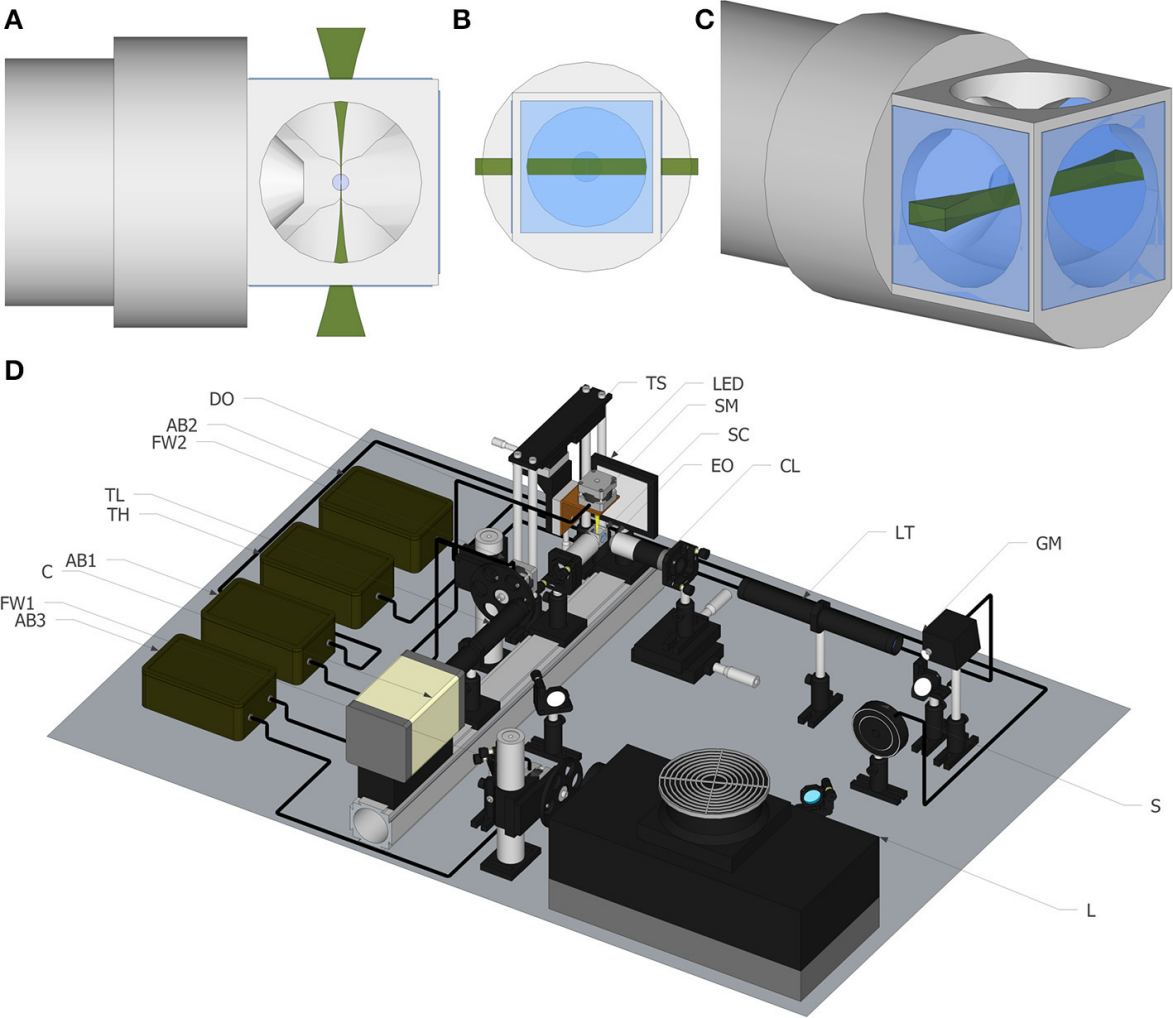
**Light sheet fluorescence microscopy. (A)** Top view of the light sheet, including objective and our newly developed chamber. **(B)** Front view of the light sheet. **(C)** Perspective view of the light sheet. **(D)** Schematic of the experimental set up. The list of elements are: Argon/Krypton laser (L), excitation filter wheel (FW1), shutter (S) galvanometric mirror (GM), excitation objective (EO), 3.5× lens telescope (LT), detection objective (DO), sCMOS camera (C), tube lens (TL), emission filter wheel (FW2), translational stage (TS), stepper motor to rotate the sample (SM), Arduino board (AB), sample chamber (SC) for water immersion objectives.

Therefore, the establishment of 3D cultures as an increasingly used strategy by the scientific community is driving the field toward the development of standardized culturing protocols and more suitable characterization and imaging techniques (Pampaloni et al., 2007). On one hand, classically used techniques for culturing cells as aggregates, such as hanging drop techniques, rotating wall vessels, and stirred culture systems, are now being explored for their potential to support the generation of accessible human 3D models (Kim et al., 2004; Justice et al., 2009). On the other hand, advances in imaging techniques will be essential to fully take advantage of these complex cultures, which are typically several hundred microns thick and highly scattering (Pampaloni et al., 2007). The quantitative analysis of the spatio-temporal organization of the different cell types in an aggregate requires well-suited 3D imaging techniques with high resolution, high speed, and minimal photodamage. However, conventional visualization techniques like point scanning or spinning disk confocal microscopes are not optimal for thick samples, providing a short penetration depth into the aggregates. This can be partially solved by performing immunofluorescence microscopy of cryosections, although limited to fixed samples. Light sheet fluorescence microscopy (LSFM) techniques have been proposed as an alternative approach, to overcome these limitations in cancer cell spheroids (Pampaloni et al., 2007; Lorenzo et al., 2011). LSFM is a fluorescence microscopy technique, where the illumination is done perpendicularly to the detection (Huisken et al., 2004; Verveer et al., 2007). The illumination laser beam is shaped into a rectangle and then focused into a thin “sheet of light” using a cylindrical lens (Selective Plane Illumination Microscopy-SPIM) or a fast moving laser scanner (Digital Scanned Light sheet Microscopy-DSLM) in the focal plane of the detection objective (Figures 1A–C). As the sample moves through the focal plane, different planes of the sample are illuminated, creating a z stack of images that can be three-dimensionally reconstructed. As the light-sheet can be tailored to the micron range, it achieves good sectioning of the sample and out-of-focus light suppression. Since illumination and detection pathways are decoupled, the lateral resolution is given by the detection objective only. Images are acquired with CCD or sCMOS cameras, enabling higher sensitivity and faster acquisition rates than photomultiplier (PMT) based devices. Also, due to inherent setup that only illuminates a fraction on the sample, it is less phototoxic when compared with conventional florescence microscopy (Reynaud et al., 2008). Moreover LSF microscopes have a good penetration depth because the numerical aperture of the illumination system is much smaller than that of the detection system. Among the several advantages the current prototypes offer, is the possibility of obtaining multi-views of the sample by rotating it, so that hidden parts of the sample become visible. This feature is not typically offered in conventional multidimensional microscopy imaging systems, such as confocal or multi-photon microscopy, and leads to the possibility of obtaining detailed 3D volume reconstruction of the sample, not achieved with any other microscopic technique.

LSFM imaging strategies offer high speeds, large fields of view and long-term imaging capacity. Here we describe the implementation of a SPIM/DSLM system and exploit its potentialities through a detailed characterization of differentiated human neural aggregates, derived from stem cells. A 3D strategy based on stirred systems was explored by our group for its potential to support CNS differentiation, resulting in a 3D CNS cell model composed of cells from the three neural lineages (neurons, astrocytes, and oligodendrocytes) (Brito et al., 2012). This has important applications ranging from drug screening to disease modeling and its coupling with the LSFM microscope developed represents one step forward in conceding the neural model system developed its full potential.

## Materials and methods

### Imaging set up description

All the images shown in this paper were acquired with a home-made light sheet microscopy system, based on an open software and hardware approach, the *OpenSpinMicroscopy* project (Gualda et al., 2013). Full descriptions of the apparatus, as well as source code of the acquisition software and instructions to build it are available through our webpage (https://sites.google.com/site/openspinmicroscopy/). The set-up of the light-sheet microscope is shown in Figure 1D. Briefly, the illumination is performed with an Argon/Kryton laser (Melles Griot 35 LTL 835-230) providing excitation wavelengths of 488, 568, and 647 nm. Excitation laser lines are selected using a filter wheel with three different filters (D488/10, 568/10 and D647/10). The laser power is controlled using a shutter (Uniblitz electronics LS3T2) and a varying neutral density filter. The laser scanning is carried in the vertical axis using a galvanometric mirror (6210H Cambridge Technologies) which optical plane is conjugated with the back focal aperture of an objective lens (Plan Fluor 4× 0.13 WD17.4 mm) using a 3.5× telescope system consisting in a 50 mm and a 175 mm plano-convex lenses. For detection, a water immersion objective (Nikon LWD 16× 0.8NA WD 3 mm or Nikon Fluor 60× 1.0NA WD 2 mm), placed perpendicularly to the excitation plane, is used to collect fluorescence emission. Excitation light is rejected using emission filters placed in infinity space before the camera, with filters mounted in a second automatic filter wheel, consisting of the following: HQ 535/70m-2p, HQ 580/25m-2p, HQ 620/90m-2p, and ET 700/75. Finally a 200 mm tube lens creates the image on the chip of a CMOS camera (Hamamatsu Orca-Flash4) recording the entire illuminated plane at the same time. With the 16× objective the total field of view is of 819 × 819 μm with a final pixel size of 0.4 μm, which means that for this objective we are actually acquiring under-sampled images.

*OpenSPINMicroscopy* provides a Java plugin for Micromanager (open-source image acquisition software for microscopy) to fully control sample rotation, filter wheels, shutter, and image acquisition as well as galvo speed and deflection angle for DSLM mode, with a single window. It allows the capture of time lapse sequences, multicolor, z stacks and multi-view recordings. The innovative approach of this setup consists on the use of cheap open source hardware, i.e., Arduino microcontrollers, opening this technology to any lab with a minimum technical background. The system uses three Arduino boards with modified firmware to control a shutter, a galvo for DSLM and three stepper motors, one for sample rotation and others for filter wheels excitation and detection. All image processing is performed using free software ImageJ.

### Sample preparation

All Human midbrain-derived neural progenitor cells (hmNPC) were differentiated as neural aggregates using stirred culture systems with orbital shaking, in the presence of morphogens and under low oxygen conditions, as previously described (Brito et al., 2012).

Transduction of differentiated neural aggregates with CAVGFP vectors (an E1-deleted CAV-2 vector expressing GFP) (Bru et al., 2010) was performed as previously described (Brito et al., 2012). Briefly, CAVGFP vectors were added to the culture, with 50% reduction of the working volume, according to the intended Multiplicity of Infection (MOI). Four hours post-transduction (hpt) the initially working volume was restored by adding fresh medium and changed at 72 hpt.

NTERA-2/cl.D1 cells (NT2) were differentiated as aggregates in 125-mL spinner vessels (from Wheaton, Techne, NJ) equipped with a ball impeller and maintained at 37°C and 5% CO_2_, as previously described (Serra et al., 2009). Briefly, neuronal differentiation was induced by addition of retinoic acid (RA, Sigma) to the culture media, at a final concentration of 10 μM. A 50% media exchange was performed three times a week for 3 weeks.

### Immunofluorescence protocol

Neural aggregates were fixed in 4% paraformaldehyde (PFA) + 4% Sucrose in PBS for 1 h at 4°C, permeabilized and blocked with 1% (w/v) Triton X-100 (TX-100) solution/0.2% fish skin gelatin (FSG) (Sigma) for 2 h at RT and subsequently incubated overnight at RT with primary antibodies (α−βIIITubulin, Millipore; α-Tyrosine Hydroxylase, Santa Cruz Biotechnology) diluted in 0.1% TX-100/0.125% FSG. Aggregates were then washed three times with 0.08% Tween 20 and incubated with secondary antibodies (AlexaFluor 488 goat anti-rabbit IgG, AlexaFluor 549 goat anti-mouse IgG, Invitrogen), diluted in 0.125% FSG, for 5 h at RT. After three washes with 0.08% Tween 20 cell nuclei were counter stained with TO-PRO-3 (Invitrogen).

### Live/dead assays

Viability of cells within differentiated neural aggregates was visualized with NucView 488 and MitoView 633 Apoptosis Kit according to the manufacturer's instructions (Biotium, Hayward, CA, USA). Briefly, neural aggregates were exposed to NucView™ 488 1× and MitoView™ 633 1× simultaneously in Hibernate (Invitogen), a media designed to maintain embryonic neural tissue in ambient CO_2_, and imaged overnight. NucView™ 488 is a caspase-3 substrat that detects caspase-3 in live cells without interfering with the enzyme activity and MitoView™ 633 is a mitochondrial dye which only stains viable cells since its fluorescence is dependent on the mitochondria's membrane potential. Tert-butyl hydroperoxide (tBHP) (Sigma), an oxidative stress inducer, was used to trigger apoptosis at a concentration of 1 mM in Hibernate. Two full stacks of images, one for each color, were acquired every 10 min during a period of 16 h, using the 16× objective and the sample mounting described below.

### Tracking of Ca^2+^ oscillations

Neural aggregates were incubated with 1× Fluo4 Direct calcium reagent (Invitrogen) for 30 min at 37°C, 5% CO_2_, and 3% O_2_ followed by 15 min at RT. Fluorescence change over time was defined as ΔF/F_0_ = (F-F_0_)/F_0_, where F is the fluorescence at any time point, and F_0_ the baseline fluorescence determined by baseline fitting across the whole movie for each cell using PeakFit Software (v4.12).

### Sample mounting

Fixed samples were embedded on 1% low melting temperature agarose and placed on the tip of a plastic pipette. The other end of the plastic pipette is inserted on the rotational stepper motor for sample rotation, which is attacked to a linear DC motor for sample scanning though the light sheet, as described in (Gualda et al., 2013). In order to perform multi-view fusion, fluorescence TetraSpeck 0.5 μm beads 1:10000 diluted (Invitrogen) are added to the agarose. The fusion is performed using the free plugin for Fiji, SPIMRegistration (Preibisch et al., 2010).

For the calcium imaging experiments, agarose was mixed with medium in order to maintain liability of the samples. Images were acquired for 30 min, and neuronal activity was still observed.

For the live/dead assay experiments samples were immersed inside Fluorinated Ethylene Propylene (FEP) tubes with a medium that ensures liability for long periods of time. FEP tubes (Kaufmann et al., 2012) are ideal for light sheet microscopy since it refractive index is close to water, reducing the optical aberrations, providing a better environment to the samples with high optical clarity. The medium, pumped with a motorized controlled syringe, can flow through the tube providing the nutrients needed by the samples to ensure liability. Samples are placed at the end of the tube, stopped by a filter that leaves the medium flow, while keeping the sample at that position. However, samples are not tidily hold so they are susceptible to move during long term experiments.

## Results

### Characterization of neural aggregates

**Figure 2:**
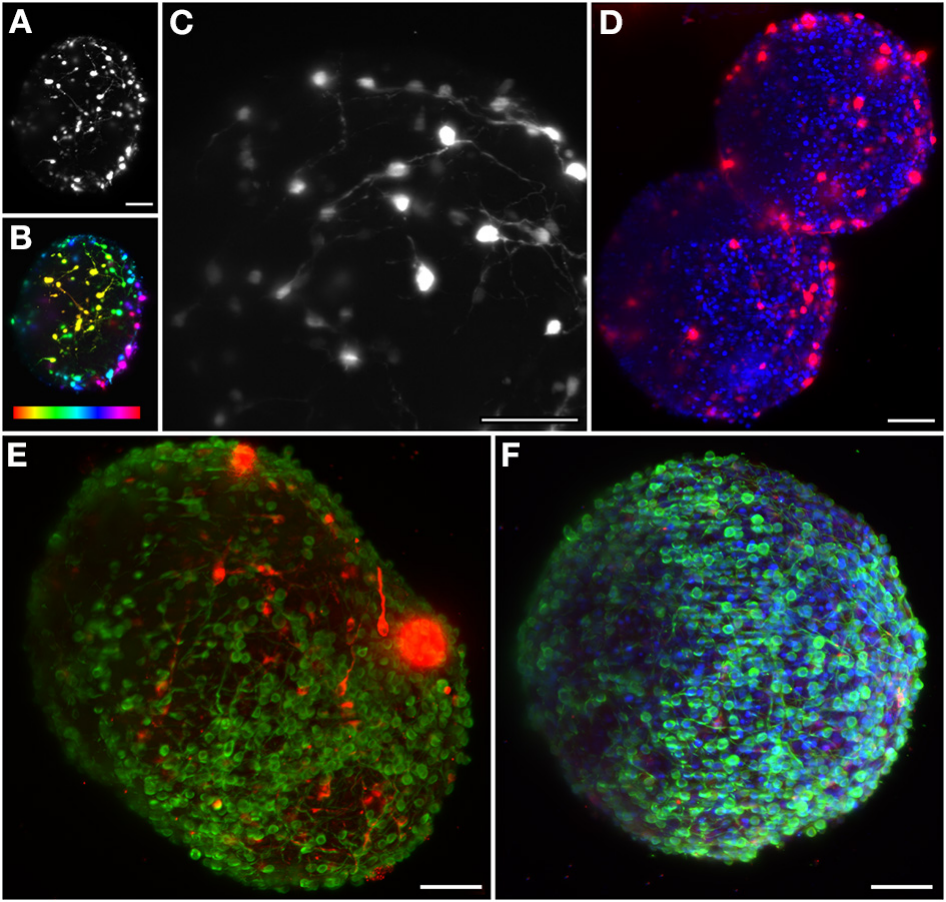
**Maximum intensity projection of different neural aggregates imaged using light sheet microscopy. (A)** Differentiated hmNPC neural aggregates expressing GFP transgene. **(B)** Same neural aggregate with depth lookup table. Red indicates 0 μm and purple 270 μm. **(C)** Transduced differentiated neural aggregate expressing GFP transgene; Objective: 60×. **(D)** Two differentiated neural aggregates with dopaminergic marker TH and TO-PRO-3 in the nuclei. **(E)** Differentiated neural aggregate with dopaminergic marker TH and bIII-tubulin was used as neuronal marker. **(F)** Differentiated neural aggregate with TH, TO-PRO-3, and bIII-tubulin. All images except **(C)** obtained with 16× objective. Scale bar: 50 μm.

The great potential of this 3D CNS cell model can be enhanced by imaging techniques that allow the study of the interaction between therapeutic viral vectors and human neural populations, within a more physiological 3D context. The model system here characterized has several applications ranging from drug screening to disease modeling (Brito et al., 2012). *In vivo* gene transfer using viral vectors is still the primary strategy for delivering novel genes to the CNS (Bjorklund and Kordower, 2010), and previous work performed by our group has proven the differentiated human neural aggregates' amenability to transduction by canine adenovirus derived vectors (CAV) (Brito et al., 2012). Our setup enables the visualization of the 3D network of the transduced cells inside the neural aggregates (Figure 2A). In order to determine the penetration depth, the same aggregate is also shown with a depth dependent lookup table. Sections on the surface are plotted in yellow, while the deepest layer is plotted in red, illustrating this network up to 270 μm deep (Figure 2B). In Figure 2C an aggregate was imaged with a 60× objective and the recorded image has been saturated to allow the visualization of neurites. Deep inside the sample, fluorescent spots are still detected, corresponding to neuronal somas, increasing the penetration depth up to 1.5-fold when compared to spinning-disk confocal microscopes (Brito et al., 2012).

**Figure 3:**
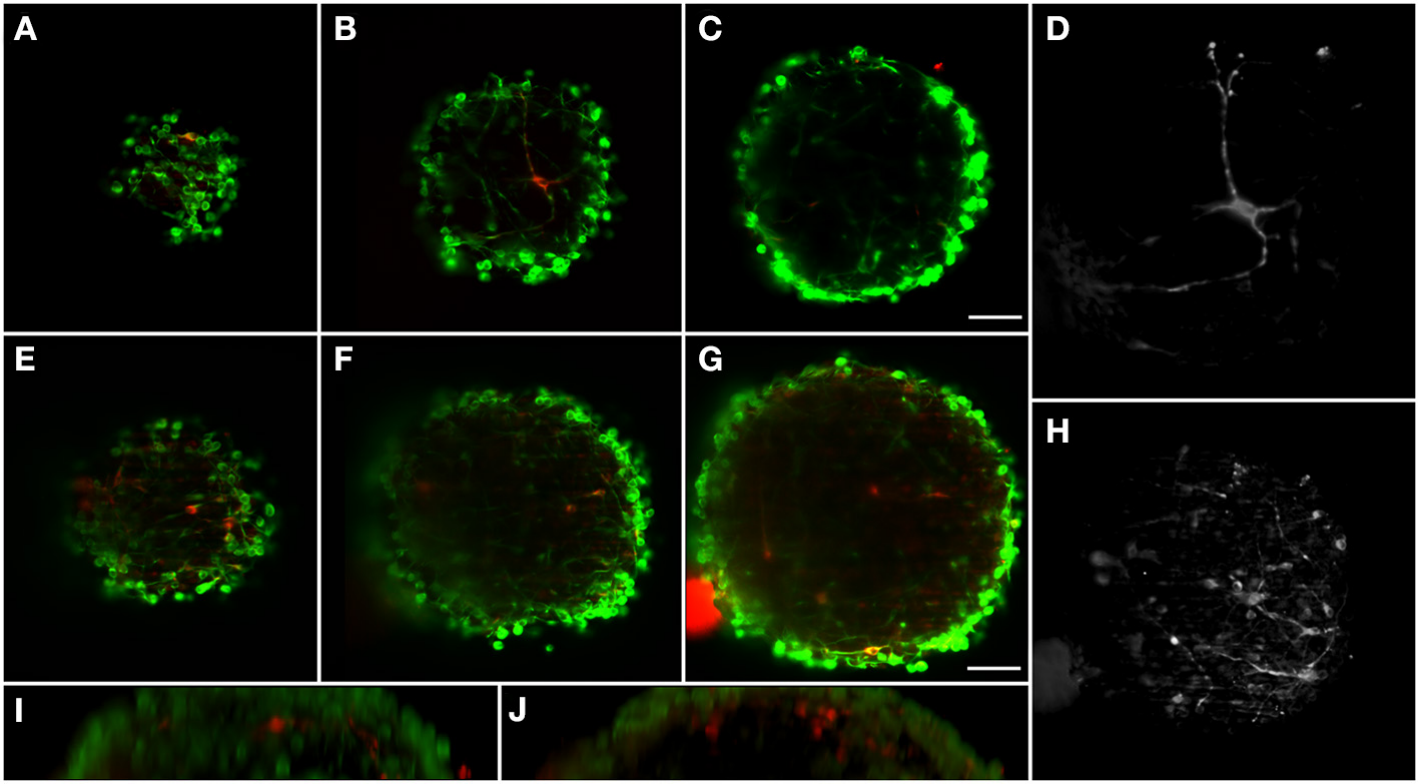
**Dopaminergic neurons distribution in two different differentiated neural aggregates with depth**. BIII-tubulin was used as neuronal marker (green) and TH as dopaminergic marker (red). Neural aggregate #1 showing mature dopaminergic neurons with long axonal connections at: **(A)** 17 μm from surface; **(B)** 43 μm; and **(C)** 75 μm. **(D)** Maximum intensity projection of the dopaminergic neuron. Neural aggregate #2 showing a complex dopaminergic neuron matrix at: **(E)** 29 μm from surface; **(F)** 59 μm and **(G)** 97 μm. **(H)** Maximum intensity projection of the dopaminergic neuronal matrix. Sagittal views of **(I)** Neural aggregate #1 and **(J)** Neural aggregate #2. A full 3D representation of the dopaminergic neurons is shown in Movie S1. Scale bar: 50 μm.

The neural circuits present in the human brain are comprised of highly diverse and specialized cell types (Nelson et al., 2006). The ability to objectively classify a specific cellular lineage is still a matter of debate and research, resulting in a growing list of phenotypic markers (Nelson et al., 2006). Therefore, the ability to simultaneously visualize different markers using multicolor staining approaches is essential when characterizing different cells in culture and studying multicellular heterotypic interactions. The light-sheet fluorescence microscope here implemented is equipped with three excitation wavelengths. In this way, we were able to optimally image three different fluorescent channels. Immunodetection of bIII-tubulin evidenced the extensive neuronal network present in differentiated neural aggregates (Figure 2E). Further investigation of the specific neuronal lineages present in culture revealed intense staining of tyrosine hydroxylase (TH), a dopaminergic marker, throughout the aggregates (Figures 2D,E). Additionally, nuclei were stained with TO-PRO-3, which provided a bright signal (Figure 2F). Taking advantage of the abovementioned increased penetration power of our set-up; the dopaminergic neuronal network present in the culture was analyzed in greater detail. TH^+^ cells were detected up to 100 μm deep within the aggregates on a single view (Figures 3A–C,E–G), indicating efficient differentiation throughout the entire neural aggregate. Figure 3H depicts the extensive dopaminergic network developed and Figure 3D highlights the long projections exhibited by these cells, suggesting their maturation and functionality (also see Movie S1). Figures 3I,J also shows that βIII-tubulin signal is lost in the deeper layers of the aggregate. Since we are still able to detect TH^+^ cells, this probably results from inefficient antibody penetration, which becomes exhausted in the first layers of cells, due to the dense neuronal network present.

### Multi-view imaging and fusion

**Figure 4:**
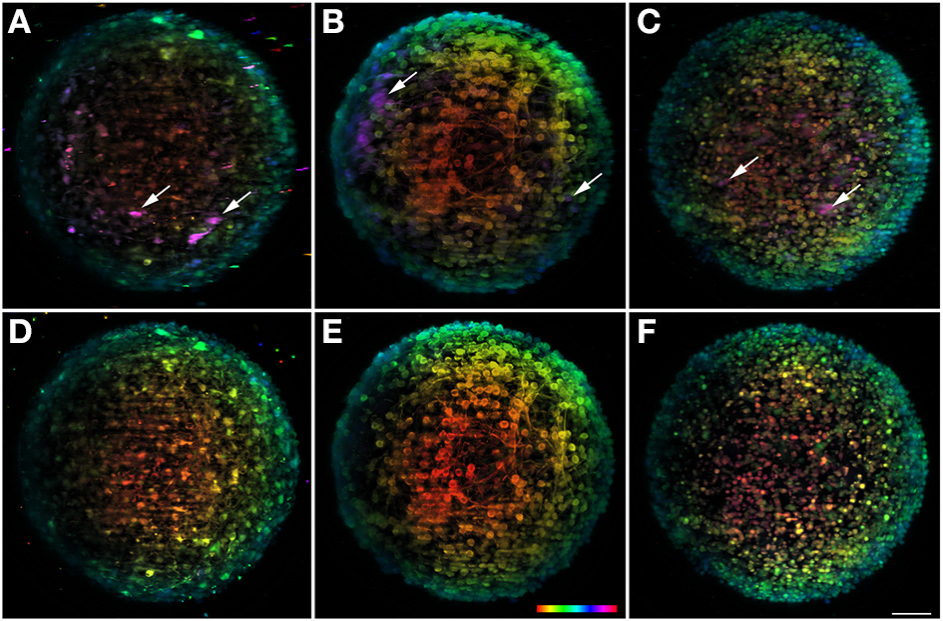
**Multi-view fusion on neuronal aggregates**. In the upper row result of the fusion of 8 stacks recorded after rotation of the sample 45° is shown for the three different channels. For the sake of comparison the lower row shows the information obtained with a single view. All images are plotted using the same depth color map. **(A,D)** Shows dopaminergic neurons with TH and **(B,E)** BIII-tubulin as neuronal marker and **(C,F)** TO-PRO-3 in the nuclei. Some neurons visible after fusion but undetectable in a single view are highlighted with arrows. Scale bar: 50 μm.

Sample rotation is one of the main characteristics of LSFM techniques and has been used to solve the problems caused by light scattering, shadowing, anisotropy, and sensitivity, inherent to the specific configuration of these microscopes (Edelstein et al., 2010). Successive z stacks can be acquired for different view angles, showing hidden features of the samples. In the case of neural aggregates, with a spherical shape and with diameters that can reach 400 μm, this feature allows imaging the center area of the sample from different angles, increasing the final penetration depth. Figures 4A–C show the maximum projection of the final fused image of TH^+^, βIII-Tubulin^+^ neurons, and TO-PRO-3 in the nuclei, respectively. To obtain the resulting image, eight single-view images recorded every 45° were computationally fused into a single 3D dataset. However, fusion is an extremely demanding process in terms of computational resources and a powerful computer with several tens of RAM memory is needed. In our case we use a HP workstation with a RAM of 128 Gb. Nonetheless, the general goal of these algorithms is to extract the “most useful” information from all the datasets and to merge it into a single dataset, replacing the inferior information that may be present in some of the datasets. Due to the computational procedure, some sharpness is lost in the final image when compared with the original data. To solve that, a free deconvolution software for Fiji recently developed can be used to increase the final quality of the image (Preibisch et al., 2014).

In order to better appreciate the multi-view information, the data set is presented with depth-dependent lookup table (Figures 4A–C), as well as the 0° single view image (Figures 4D–F). When comparing the two set of images, the amount of information that is gained with the multi-view fusion becomes clear. Specifically, there is a considerable number of undetectable neurons within the single view (Figure 4; arrows), that become visible with the multi-view fusion. This shows the potential of the rotating feature, unique to this microscope, for extracting more information from the sample and for providing a more accurate analysis of the object in study. The volume reconstruction of some neurons can be better observed on the Movie S2.

### Tracking of Ca^2+^ oscillations

**Figure 5:**
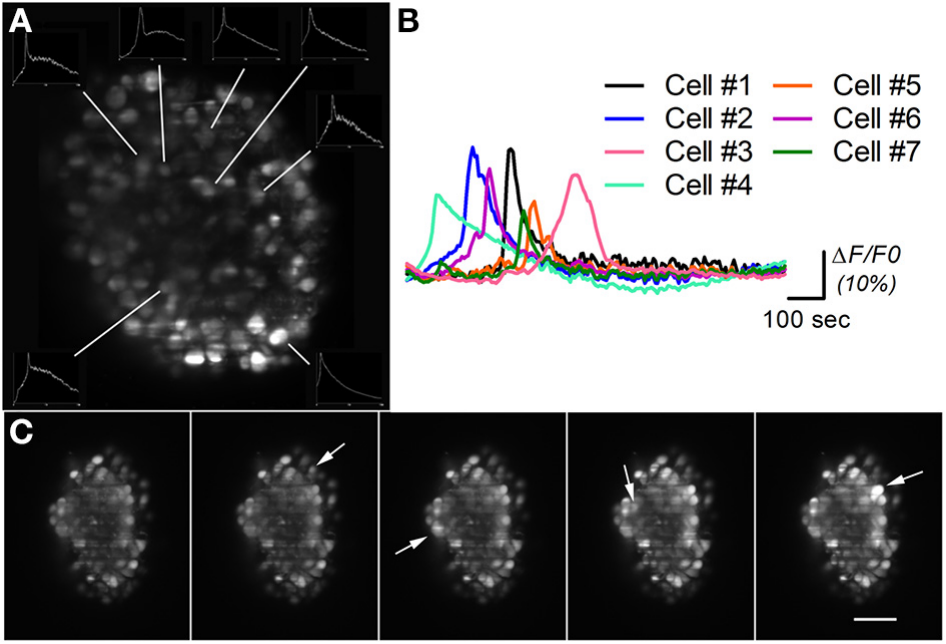
**Calcium imaging on differentiated neural aggregates stained with AM-ester calcium indicator. (A)** Section of a neural aggregate with Ca^2+^ oscillations (insets) **(B)** Normalized intensity of the Ca^2+^ oscillations at seven different cells of neural aggregate in **(A)** imaged during 25 min. **(C)** Five time points of a neural aggregate showing Ca^2+^ oscillations in different cells (arrows). A time lapse movie is shown in Movie S3. Scale bar: 50 μm.

Having demonstrated the utility and advantages of this system for fixed samples, its potentialities for *in vivo* imaging were explored through a set of experiments on calcium imaging for assessment of neural cells' functionality. Differentiated neural aggregates were exposed to the Fluo4 live probe, an AM-ester calcium indicator. Loaded samples were embedded in agarose and imaged during 15–30 min. Movie S3 shows a movie recorded during 25 min on two neural aggregates. Every image is composed by the maximum projection of five different layers, two microns apart (represented in a different color) covering a thickness of 10 microns, acquired every 6 s. The monitoring of fluorescence intensity over time enabled the detection of spontaneous Ca^2+^ oscillations within the cells in culture, which differed in frequency, regularity, and duration (Figures 5A,B). After this time the activity ceases since at the time of acquisition the system did not have a proper incubation chamber with CO_2_ control. Nevertheless, this technique allowed recording different planes in a fast way, enabling the reconstruction of the 3D map of spontaneously occurring transients in the intracellular Ca^2+^ concentration in neural cells.

Figure 5A shows a section of a neural aggregate stained with AM-ester calcium indicator. Ca^2+^ oscillations were observed in seven different cells during the 25 min of observation. The insets in Figure 5A show the total intensity over time, recorded on the area surrounding the analyzed cell. The normalized recorded signal is plotted in Figure 5B, as described in the experimental procedures. Different time points of a different neural aggregate where cells pointed by arrows shows an increase of fluorescent signal are displayed in Figure 5C. During the period of observation conformational changes in the structure were observed, as well as changes in the overall fluorescence. Same results have been obtained with a spinning disk confocal microscope (Simão et al., under review). The advantage of a LSFM consist that same type of results can be obtained in a system more than one order of magnitude cheaper, with high speed, able to simultaneously record the activity of the cells in the surface, as well as the deeper layers of the neural aggregate. Although high speeds, up to 100 fps in our system, can be acquired on a single slice, 3D imaging of neural activity is limited to 4 fps by the slow movement of the translational stage for sample scanning, as discussed below.

### Live/dead assays

**Figure 6:**
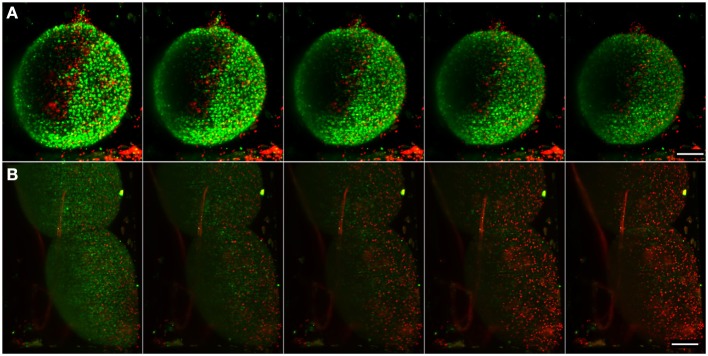
**Imaging of apoptosis in differentiated neural aggregates**. Maximum intensity projection of five different time points, acquired every 150 min, of samples with NucView 488 (green) and MitoView 633 (red). **(A)** Control sample with only medium. **(B)** Differentiated neural aggregates exposed to tBHP, which triggers apoptosis. Movies S4, S5 show the correspondent movies over a period of 16 h, acquiring a full stack every 10 min. Scale bar: 100 μm.

The great potential for the 3D CNS cell model presented to increase the reliability of pre-clinical trials is dependent on the development of suitable characterization techniques. To test our system's potential to address this question, we performed a set of experiments on real-time analysis of apoptosis. Differentiated neural aggregates were kept in the field of view in a medium, as described in the experimental procedure section, that allows proper maintenance of neuronal cells in ambient CO_2_ and temperature conditions, exposed to NucView™ 488 Caspase-3 Substrate and MitoView™ 633 mitochondrial dye, and imaged for 16 h recording a two-color stack every 10 min. The samples remained viable for the duration of the assay (Figure 6A and Movie S4), with the majority of the cells viable, apart from the few initial apoptotic cells typically present in these cultures. These results indicate that the setup is suitable for performing toxicity experiments, as the phototoxicity resultant from long-term imaging is low enough to have no impact on apoptosis.

Using the setup described above, differentiated neural aggregates were exposed to tBHP, a potent oxidant, which triggers apoptosis (Peterson et al., 2007). Figure 6B and Movie S5 shows the toxicant effect over time. Using apoptosis and mitochondrial activity as readouts, as opposed to the usually conducted end-point toxicological assays, our system enables a dynamic, real-time measuring of apoptosis. At the concentration of toxicant tested, cells remained viable for approximately 16 h. Apoptotic cell death occurs at time-point 40 (after 6 h of imaging) and is widespread through the neural aggregate, indicating equivalent sensitivity to the toxicant of the deeper layers of cells. As a proof of concept, these results show that the inherent low phototoxicity of the system here developed, coupled with different fluorescent probes, allows the assessment of different levels of metabolic activity in cells in normal conditions and in response to a given compound, increasing the reliability of pre-clinical studies.

## Discussion

LSFM imaging techniques are useful to image broad collection of fixed differentiated human neural aggregates, with different immunofluorescence labeling, as well as live imaging. The DSLM configuration is able to provide a complete view of the neuronal network of βIII-Tubulin^+^ cells present in these samples. The possibility to rotate the object in different directions, shining light into otherwise inaccessible areas of the sample and increasing axial resolution gives us the overall picture and better insight into the structures in study. Also, this setup allows for multichannel sequential acquisition. Our results demonstrated that with SPIM/DSLM system's increased penetration depth over widely used confocal microscopes we are able to spatially resolve the inner morphology of the dopaminergic networks and detection of GFP^+^ cells, after CAVGFP transduction, in the inner layers of the aggregate. We developed a versatile SPIM/DSLM system based on open software (Micromanager) (Edelstein et al., 2010) and hardware (Arduino boards for device control), providing a specialized plugin to image acquisition control, including sample rotation, a feature that is not available in standard systems. Our open source platforms, OpenSpinMicroscopy (Gualda et al., 2013), is available through a dedicated webpage for custom construction of light-sheet microscopes, making these systems available to the labs with minimum background. LSFM techniques provide a powerful tool for imaging thick complex samples, including long term *in vivo* imaging experiments, because their special configuration and advance capacities overcome the classical limitation shown by traditional single axis illumination microscopy techniques, increasing speed and penetration while reducing bleaching and phototoxicity. Previous works have already demonstrated the capabilities of LSFM in 3D cell cultures (Lorenzo et al., 2011; Bruns et al., 2012). Madin-Darby canine kidney (MDCK) cysts grown in extracellular matrix hydrogels have been successfully imaged by SPIM microscopes (Reynaud et al., 2008; Swoger et al., 2014). Moreover, monitoring of live cell division dynamics in 3D large multicellular tumor spheroids (HCT116 colon carcinoma) expressing a histone H2B-HcRed fluorescent nuclear reporter protein has been recorded (Lorenzo et al., 2011).

Although those works have shown the great achievement on tumor cancer cells, to our knowledge, this is the first time that light-sheet microcopy techniques has been applied for imaging of neural aggregates. The study of the human CNS is especially challenging due to its anatomical and functional complexity. Neuronal signal transduction and communication *in vivo* depends on highly complex and dynamic 3D networks among neurons. Studies of cell-cell and cell-matrix interactions, synaptogenesis, and neural network plasticity are pivotal in improving our understanding of neurodegenerative disorders, such as Parkinson's disease. 3D cell based models are thus becoming the widespread strategy used to address these issues. However, to fully take advantage of these models and study cells' phenotype, interaction, and spatial organization within the aggregates, there is a need for more powerful and informative tools that enable a characterization *in toto* of the culture with sufficient resolution in space and in time (Pampaloni et al., 2013). The large size and highly scattering nature of these samples constitutes a challenge when it comes to imaging and classical imaging techniques do not seem to be ideal in terms of penetration depth and rapid acquisition. We believe that the LSFM system presented here represents an important breakthrough in order to fully exploit the benefits of the third dimension in the life sciences, and particularly in neural cell biology. Our major interest at this point is the improvement of the sample preparation protocol for efficient immune-staining of the inner part of the aggregates to further test the penetration depth of our set-up. Nevertheless, with the use of cells expressing constitutive fluorescent markers, we are able to bypass the limiting step of antibody penetration and fully exploit the potentialities of the system. Our results demonstrate this setup's ability to visualize the 3D network of cells transduced with viral vectors. This highlights the potential that arises from combining this powerful imaging technique with 3D cell models for future studies on the efficacy of viral vectors as gene delivery approach.

The development of new compounds targeting CNS disorders has struggled with toxicity and efficacy issues during clinical trials, with only an 8% approval rate in clinical trials (Miller, 2010). Moreover, the failure of the drugs tends to happen in later stages of the clinical trials, resulting in huge investment losses (Miller, 2010). Therefore, new approaches are needed to screen and characterize the neurotoxic potential of chemicals in pre-clinical stages.

LSFM specific configuration, with the use of fluorescence optical sectioning, exposes the samples up to 5000 times less energy than commonly used confocal microscopes, making it suitable for imaging live cultures over long periods of time, with reduced photobleaching and phototoxicity (Pampaloni et al., 2013). As proof-of-principle, we developed a dedicated incubation and feeding systems based on FEP tubes, where we are able to avoid the use of agarose and maintain live cells under conditions of higher physiological relevance to perform long-term toxicity studies with the use of fluorescent probes. Our results demonstrate that this setup is able to maintain differentiated neural aggregates viable for the duration of the assay. The readily accessible feeding tube gives the possibility to add nutrients, pharmaceutical agents and fluorescent dyes without moving the samples, and enables real time acquisition and monitoring of the effects. In this study, a prototypical stress inducer drug was used—tBHP (Peterson et al., 2007)—and time-dependent cell death by apoptosis was monitored. With this setup and the use of an apoptotic fluorescence marker we were able to surpass the usually conducted endpoint assays and visualize the toxicant compound effects in real time, as well as to follow-up of each individual sample along time.

We have demonstrated that our setup is able to deliver a spatially resolved analysis of new therapeutics effects in 3D models. By combination with different fluorescent probes, this system has the potential to undercover a given compound's outcome at the morphological, biochemical and gene expression levels, highly improving the accuracy of toxicity screenings in pre-clinical trials.

Because of their high quality (especially good axial resolution, high dynamic range, and low noise), LSFM datasets generally do not require common preprocessing routines such as denoising, deconvolution, or unmixing. Rather, it is the complexity and quantity of the new information acquired that calls for new tools for data segmentation, visualization and navigation. As SPIM data are mostly not only 3D, multi-color and multi-view, but also represent a time sequence, it is especially challenging to extract and illustrate the dynamics that are hidden in these data. We have shown how multiview fusion in fixed samples can be useful to obtain information that otherwise will be lost, on traditional microscopy techniques lacking sample rotation. However there are still limitations inherent to the new way of seeding light into the sample can be easily overcome increasing the complexity of the system. One of the major limitations of LSFM is the creation of stripe patterns due to absorption of light at the surface of the samples on the light propagation path. Different approaches have been proposed to overcome those artifacts. On one hand image processing for variational stationary noise removal (Fehrenbach et al., 2012) or multi-view fusion (Preibisch et al., 2010), used in this work, allow to correct those artifacts up to some extent, but requires powerful workstations and long processing times. On the other hand double side illumination systems (Huisken and Stainier, 2007) or systems that allow to pivot the light sheet (Greger et al., 2007) remove stripes of shadows generated by the light being scattered in the first layers during acquisition, reducing the image processing time. Another promising approach is by the used of self-regenerative Bessel laser beams (Olarte et al., 2012). Image acquisition frame rate is another issue that must be taken into account, depending on the processes under analysis. Nowadays, sCMOS cameras allow to record at higher frame rates, so the limitation on the acquisition speed depends only on the fluorescent signal of our sample, and the sample scanning speed. Our system is actually limited, for 3D imaging, by the scanning method, where the sample is moved through the light sheet at relatively slow speeds, allowing a maximum of four frames per second at full resolution. Recently different approaches, where the sample is maintained at the same position, while the focal point of the detection objective is moved in coordination with the light sheet axial position, allowed increased imaging speeds. The position of the objective focal point can be modified either mechanically with a piezo-electric motor such the case of the iSPIM system (Wu et al., 2011) or optically using tunable lens (Fahrbach et al., 2013) providing from rates from 200 to 500 fps. However those systems do not include the sample rotation option. Moreover, configuration with two sided illumination and two cameras increase the speed of the system either by imaging two views simultaneously, so reducing the number of views needed for image fusion either acquiring two fluorescence channels simultaneously (Krzic et al., 2012). Finally, another limitation of this technique for experimental application is the fact that samples need to be mounted on agarose one by one in order to be imaged, limiting the number of samples analyzed. High throughput imaging could represent the last barrier, since sample mounting by embedding it in agarose limits the controlled delivery of drugs or an ideal environment for long term imaging, such as cell proliferation and differentiation. However new advances has been presented for combining light sheet imaging approaches to microfluidics (Bruns et al., 2012), flow cytometry (Wu et al., 2013), and tissue culture experiments (Ansari et al., 2013), opening the door to increasingly complex experiments.

3D models are progressively becoming more relevant as research tools and consequent advances in optical microscopy are required to more accurately assess these thick and highly scattering samples. This 3D organization is especially important in the CNS, where the connections between cells actively contribute to their maturation and functionally (Momčilović et al., 2012). Therefore, light sheet-based fluorescence microscopy is here presented as a promising approach, not only to assess immunofluorescence staining of fixed samples, but also suitable to monitor functional properties of the different neural cells in live assays.

### Conflict of interest statement

The authors declare that the research was conducted in the absence of any commercial or financial relationships that could be construed as a potential conflict of interest.

## Supplementary material

Movie S1 **3D reconstructions of two dopaminergic neuronal networks stained with the dopaminergic marker tyrosine hydroxylase (TH) showing their maturation and functionality**.

Movie S2 **Volume reconstructions of 3D neural aggregates**. Left 3D volume corresponds to the information recorded on a single view while the right 3D volume to the obtained after the fusion of 8 different views 45° apart.

Movie S3 **Tracking of the Ca^2+^ oscillations in two neural aggregates stained with AM-ester calcium indicator Fluo-4 over 25 min every 6 s**. Here is represented the maximum intensity projection of five different planes 2 μm apart, plotted with different color.

Movie S4 **Control test for long term sample liability**. Time lapse movie over 16 h of a neural aggregates stained with the apoptosis kit NucView™ 488 Caspase-3 Substrate (green) and MitoView™ 633 mitochondrial dye (red).

Movie S5 **Induced cell death with Tert-butyl hydroproxide (tBHP)**. Time lapse movie over 16 h of a neural aggregates stained with the apoptosis kit NucView™ 488 Caspase-3 Substrate (green) and MitoView™ 633 mitochondrial dye (red).
